# Metacognition of Working Memory Performance: Trial-by-Trial Subjective Effects from a New Paradigm

**DOI:** 10.3389/fpsyg.2016.00927

**Published:** 2016-06-21

**Authors:** Andrew C. Garcia, Sabrina Bhangal, Anthony G. Velasquez, Mark W. Geisler, Ezequiel Morsella

**Affiliations:** ^1^Department of Psychological and Brain Sciences, University of DelawareNewark, DE, USA; ^2^Department of Psychology, San Francisco State UniversitySan Francisco, CA, USA; ^3^Department of Neurology, University of California, San FranciscoSan Francisco, CA, USA

**Keywords:** working memory, cognitive control, consciousness, metacognition, urges to err

## Abstract

Investigators have begun to examine the fleeting urges and inclinations that subjects experience when performing tasks involving response interference and working memory. Building on this research, we developed a paradigm in which subjects, after learning to press certain buttons when presented with certain letters, are presented with two action-related letters (the memoranda) but must withhold responding (4 s) until cued to emit the response associated with only one of the two letters. In the Congruent condition, the action corresponds to the cue (e.g., memoranda = AB, cue = B, response = B); in the Incongruent condition, the action corresponds to the other item of the memoranda (e.g., memoranda = AB, cue = B, response = A). After each trial, subjects inputted a rating regarding their subjectively experienced “urge to err” on that trial. These introspection-based data revealed that, as found in previous research, urges to err were strongest for incongruent trials. Our findings reveal, first, that subjects can successfully perform this new task, even though it is more complex than that of previous studies, and second, that, in this new paradigm, reliable subjective, metacognitive data can be obtained on a trial-by-trial basis. We hope that our novel paradigm will serve as a foundation for future experimental projects on the relationship between working memory performance and consciousness—an under-explored nexus whose investigation is likely to reveal insights about working memory, cognitive control, and metacognition.

## Introduction

Today, investigators have begun to examine the fleeting ‘urges,’ ‘inclinations,’ and ‘tendencies’ that subjects experience when performing tasks involving response interference and working memory (Mayr et al., 2003; Mayr, 2004; Morsella, 2005; Mulert et al., 2005; Rosen et al., 2007; Corallo et al., 2008; Morsella et al., 2009b)^1^. Evidence suggests that these subjective effects are not ephemeral or capricious but systematic, reliable, and capable of being predicted by theoretical frameworks. In this article, we first review in brief the literature on trial-by-trial, subjective (metacognitive) effects that arise in experimental paradigms involving cognitive control, response interference, and working memory. Afterward, we introduce a new behavioral paradigm that builds on this prior research and that could be used to investigate the subjective effects of working memory performance on a trial-by-trial basis.

### Metacognitive, Subjective Aspects of Cognitive Control during Action Production

Concerning response interference, for example, investigators have examined the trial-by-trial subjective effects that occur in the classic Stroop task (Stroop, 1935). (We discuss the Stroop task because subjective variants of the paradigm serve as the basis of the present project involving working memory.) In this task, subjects are instructed to name the color in which a word is written. When the word and color are incongruent (e.g., RED presented in blue), response conflict leads to increased error rates, response times (RTs), and reported urges to make a mistake (Morsella et al., 2009a). (To obtain the “urges to err” measure, after each trial, subjects were presented with, “How strong was your urge to make a mistake?”, which they rate on an eight-point scale, in which 1 signified “almost no urge” and 8 signified “extremely strong urge.”) It has been proposed that, in the incongruent condition, there is conflict between word-reading and color-naming plans (Cohen et al., 1990). When the color matches the word (e.g., RED presented in red), or is presented on a neutral stimulus (e.g., a series of x’s as in “XXXX”), there is little or no interference (see review in MacLeod and MacDonald, 2000).

The Stroop task possesses a limitation: the incongruent conditions cannot be used to distinguish the effects of interference occurring at different stages of processing (e.g., at perceptual-semantic levels or response selection levels). The Eriksen flanker task (e.g., Eriksen and Schultz, 1979) was developed to address this issue. It reveals that introducing interference at different stages of processing (e.g., perceptual versus response selection) leads to distinct behavioral, neural, and subjective effects (Coles et al., 1985; van Veen et al., 2001; Morsella et al., 2009b). In one version of the task, subjects are trained to press one button with one finger when presented with the letter S or M and to press another button with another finger when presented with the letter P or H. Subjects are then instructed to respond to the stimulus presented in the center of an array (e.g., SSPSS, SSMSS, targets underscored) and to disregard the flanking distractors. RTs and self-reported, trial-by-trial ‘urges to err’ are greater when distractors are associated with a response that is different from that of the target [*response interference* (RI); e.g., SSPSS] than when the distractors are different in appearance but associated with the same response [*perceptual interference* (PI); e.g., SSMSS; Morsella et al., 2009b], a difference attributed to the automatic activation of response codes by distractors (Eriksen and Schultz, 1979; Coles et al., 1985; Mattler, 2005). Responses are fastest when flankers and targets are identical (e.g., SSSSS).

The pattern of subjective effects found in the Stroop and flanker tasks is found also in other *response interference* paradigms (see review of other paradigms in Morsella et al., 2011). In general, stronger subjective effects (e.g., urges to err) are systematically associated with experimental conditions featuring high levels of response interference. Specifically, *when response interference is low or absent, urges to err and perceptions of competitions tend to be low while perceptions of control tend to be high; when response interference is high, urges to err and perceptions of competition tend to be high while perceptions of control tend to be low* (Morsella et al., 2009b, 2011).

One interesting circumstance is the Stroop congruent condition. That trial-by-trial urges to err are low for this condition is interesting because it is known that, in this situation, subjects often do read the stimulus word inadvertently: “The experimenter (perhaps the subject as well) cannot discriminate which dimension gave rise to the response on a given congruent trial” (MacLeod and MacDonald, 2000, p. 386). (See treatments of the Stroop congruent condition in Eidels et al., 2010 and Roelofs, 2010.) Importantly, urges to err for the congruent condition are comparable to those of the ‘neutral’ condition of the Stroop task, in which the color is presented on an illegible letter string (Morsella et al., 2009b). It has been found that ‘urges to err by reading the word’ are greater when words are presented in standard black font than when the same words are presented in a congruent color (Molapour et al., 2011), suggesting that the act of color-naming masks introspection of the reading process which may be occurring automatically. This finding has been explained as an instance of *synchrony blindness*, in which one is unaware that two distinct cognitive operations are activated when the operations lead to the same action plan (Molapour et al., 2011). This notion is consistent with the view that one is conscious only of the ‘outputs’ of processes, not of the processes themselves (Lashley, 1951). (See review of this notion in Morsella and Bargh, 2010.)

The pattern of results reviewed above, in which incongruent conditions yield response interference effects and congruent conditions yield synchrony blindness has been explained by theoretical developments about the primary function of conscious/controlled processes in action production (Sanders, 1983; Pashler, 1995; cf. Morsella, 2005; Morsella et al., 2011). Importantly, one of these theoretical developments (Morsella, 2005) was intended to explain a different class of phenomena, such as why skeletomotor action conflicts (e.g., holding one’s breath) reliably perturb consciousness but other kinds of conflicts, such as intersensory conflicts (e.g., the McGurk effect; McGurk and MacDonald, 1976) and conflicts involving smooth muscle, do not (for further explanation, see General Discussion and Morsella, 2005; Morsella et al., 2009a, 2011).

These trial-by-trial subjective effects are unlikely to reflect solely an artifact of subjects observing their own RTs, for the effects arise even when the influence of RTs is statistically accounted for, and also when RT effects are eradicated by having subjects delay their response for a substantial span (Morsella et al., 2009b). Moreover, though subjects’ post-error corrections in interference paradigms lead, on a subsequent trial, to improved performance (e.g., faster RTs), reported urges to err actually increase in such trials (Etkin et al., 2010; Gyurak et al., 2011). This contrast has been explained as a dissociation between implicit measures of performance (e.g., RT) and explicit measures (e.g., self-reports about task difficulty; Etkin et al., 2010; Gyurak et al., 2011).

### Metacognitive, Subjective Aspects of Control during Working Memory Performance

The findings from the Stroop and flanker tasks stem from paradigms in which targets and distractors are visually available as stimuli that are present in the external environment. However, in everyday life, seldom is it the case that planned action is driven wholly by representations that are activated by stimuli in the current environment. Many actions, such as goal-directed actions, are guided by representations that are generated internally (Miller et al., 1960; Neisser, 1976). These goal-directed actions include voluntarily holding one’s breath, searching for one’s car keys, or holding a telephone number in mind. These tasks require that representations be held actively in mind with minimal aid from the external environment and even in the presence of distracting, external stimulation. These acts usually involve *working memory*, which has been defined as a temporary, capacity-limited storage system under attentional control, used to intentionally hold and manipulate information (Baddeley, 1986, 2007).

Theorists have long noted that working memory is intimately related to conscious processing (Gray, 2004; Baddeley, 2007; LeDoux, 2008; Oberauer and Hein, 2012). It might well be that no mental operation is as consistently coupled with conscious processing as is working memory (Baddeley, 2007; LeDoux, 2008): when one tries to hold or manipulate information that is not furnished by the external world, one’s conscious mind seems to be occupied with the task at hand (James, 1890). This occurs, for example, when one holds a to-be-dialed telephone number in mind and mental imagery occupies one’s conscious mind till the number is dialed (Paivio, 1979). Although many sophisticated processes can be carried out unconsciously, the deployment of working memory tends to be a conscious phenomenon (but see Hassin, 2005).

In one experiment involving working memory (Hubbard et al., 2013), subjects were instructed to hold two stimuli (e.g., the ‘S’ and ‘P’ of the flanker task) in mind until a cue prompted them to respond to one of the two stimuli; in another version (Hubbard et al., 2013), subjects were instructed to respond to the letter in the center of the screen (the target) but to delay responding until presented with a subsequent letter (the distractor), with subjects instructed to disregard the characteristics of distractors and emit only the response associated with the target. (It is important to note that, in this experiment, there was a delay period between the target and the distractor.) This working-memory version of the flanker task led to the same kinds of subjective effects associated with the RI and PI conditions of the traditional version of the task (for similar findings, see Jantz et al., 2013).

Relevant research (Jantz et al., 2013, 2014; see also Kroll and Kellicutt, 1972) reveals that the process of rehearsal during the use of working memory produces mental imagery of the memoranda throughout the delay period. Specifically, the investigators examined the subjective effects of (*a*) holding in mind information having a low versus high memory load, and (*b*) holding memoranda in mind during the presentation of distractors (e.g., visual stimuli associated with a response incompatible with that of the memoranda). The data revealed that higher rates of rehearsal (conscious imagery) occurred in the high load and distractor conditions than in comparable control conditions. Examination of the temporal properties of the rehearsal-based imagery revealed that imagery events occurred evenly throughout the delay period. The imagery is experienced as repetitive and as punctate (i.e., discrete), just as the lyrics from a subvocalized song are experienced during a span but only one word at a time. When such imagery is intentional and not an involuntary “earworm,” the imagery must be activated effortfully (Farah, 2000).

Limitations of such working memory experiments is that, in many tasks, (*a*) subjects know which response to execute when presented with the “go” cue, (*b*) subjects know which information from the memoranda will be action-relevant, and (*c*) the association between the retrieval cue and the part of the memoranda that must be acted upon is straightforward. Such characteristics are not found in many everyday tasks involving working memory. For example, regarding *a* and *b*, one might hold memoranda in mind but be uncertain regarding which memorandum will, in a given context, be action-relevant. For instance, when shopping for groceries, though one might hold in mind a list of many food items (e.g., vegetables, fruits, and meats), at one moment in time and in a particular context, one might act with respect to only a subset of the items. Such a constraining context might be that one is in the vegetable section or hearing an advertisement stating that only fruits, of all things on the shopping list, are on sale. The cues from such contexts cause one to respond to only a subset of the tokens composing the memoranda. Regarding *c*, it is often the case that a retrieval cue signifies, not that one should act in a manner corresponding to the memorandum that is associated with that cue, but that one should act in a manner corresponding to some other component of the memoranda. For example, if the memoranda consists of the tokens *X* and *Y*, the cue *Y* could signify that one should respond, not to *Y*, but to *X*. Experiments focusing on the subjective aspects of working memory performance have not yet yielded data from such circumstances.

To deal with this limitations and gap in the literature, we developed a new behavioral paradigm in which subjects, after learning to press certain buttons when presented with certain letters, are presented with two action-related letters (the memoranda) but must withhold responding (4 s) until cued to emit the response associated with only one of the two letters. In the Congruent condition, the action corresponds to the prompt (e.g., memoranda = AB, prompt = B, response = B); in the Incongruent condition, the action corresponds to the other item of the memoranda (e.g., memoranda = AB, prompt = B, response = A). After each trial, subjects inputted a rating regarding their urge to err. This was our primary dependent measure.

Our primary aim was to develop a new experimental paradigm that involves working memory and yields subjective, metacognitive data on a trial-by-trial basis. We strove to develop a task that subjects can perform even though (*a*) they do not know which response to execute before being presented with a go cue, (*b*) they do not know which information from the memoranda will be action-relevant, and (*c*) the association between the retrieval cue and the component(s) of the memoranda to be acted upon is not straightforward, requiring the application of a complex rule held in prospective memory^2^.

Regarding our task, subjects responded by pressing one of two keys with either their index finger or middle finger (as in the flanker task; Eriksen and Schultz, 1979). To diminish spatial compatibility effects (e.g., the Simon effect; Simon et al., 1970) stemming from such a left/right response mapping, the memoranda was presented, not with one letter to the side of the other, but with one letter above the other. In this paradigm, for the Incongruent condition, subjects cannot simply apply the strategy of always pressing the key opposite to that of the prompt, because of catch trials in which the memoranda consists of two identical items (memoranda = AA or BB). Catch trials, in which the memoranda consisted of two identical letters, composed half of the trials.

It could be that, in the standard (non-catch) trials of the Incongruent condition, the prompt induces interference only because it primes motorically a response that is incongruent with the intended responses. To examine this methodological limitation and for the sake of comparison, we included another condition in which the prompt was not a letter (a dot, as in Hubbard et al., 2013), but was associated with the spatial location of a target; in the Incongruent condition, subjects responded to the item that was not cued spatially.

Our new paradigm builds incrementally on previous research (e.g., Eriksen and Schultz, 1979; Hubbard et al., 2013). Thus, it yields the kind of incremental research that is important for progress in the field of psychological science and that does not involve the traditional method of hypothesis testing (see General Discussion and Nosek et al., 2012). Because it is a new paradigm, our aims and predictions had to be humble and conservative. Again, our primary aim was to assess whether subjects are capable of performing this task. Our secondary aim was to assess whether the task could yield the kinds of subjective effects that have been found with previous studies (e.g., Hubbard et al., 2013; Jantz et al., 2013). Such effects would stem from the factor Congruence (i.e., congruent versus incongruent trials): we predicted that, on average, urges to err would be stronger for incongruent than congruent trials.

For our primary aim and to develop and refine the new paradigm, we first conducted a pilot study to assess whether subjects can perform this task, which is more complicated than those of previous studies (e.g., Hubbard et al., 2013).

## Pilot Study

### Method

#### Subjects

San Francisco State University students (*n* = 29) participated for course credit. The involvement of human subjects in our project was approved by the Institutional Review Board at San Francisco State University.

#### Stimuli and Apparatus

The stimuli for the memoranda consisted of two letters (A and B), which were separated by a horizontal bar, thereby resembling the presentation of a fraction (e.g., A over B, or A/B). The letter pair was displayed within a centered visual angle of 4.18° × 6.56° (3.5 cm × 5.5 cm). The horizontal bar was 3.5 cm wide. The positioning of the letters, along the vertical axis, was counterbalanced fully across the trials. For the Letter Prompt condition, the prompt was one of these letters. For the Dot Prompt condition, the stimulus was a filled circle (●). Both kinds of prompts were displayed within a centered visual angle of 2.98° × 4.18° (2.5 cm × 3.5 cm). All stimuli were presented on a computer monitor (50.8 cm) with a viewing distance of approximately 48 cm. All questions and instructions were presented in san serif black font (~36-point) on a gray background. Stimulus presentation was controlled by SuperLab Software. Catch trials, in which the memoranda consisted of two identical letters, composed half of the trials.

#### Procedures

**FIGURE 1:**
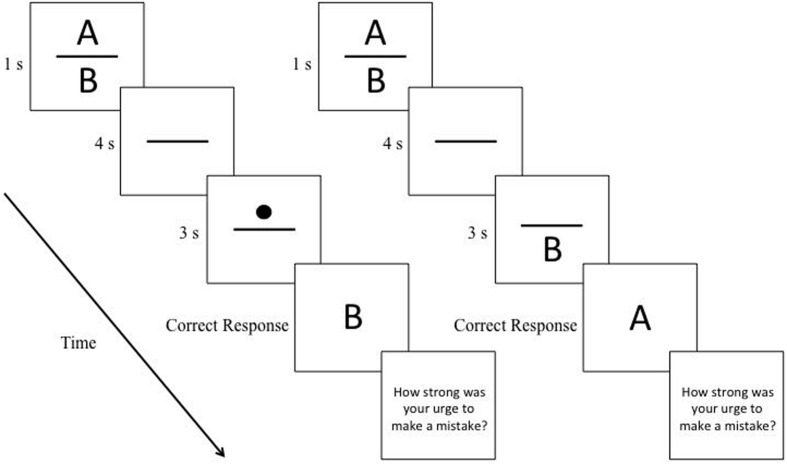
**Left: sequence of events during an incongruent trial having a dot prompt (see Pilot Study); Right: an incongruent trial having a letter prompt (see Experiment).** Not drawn to scale. Subjects were presented with two action-related letters (the memoranda) but delayed responding (4 s) until cued to emit the response associated with only one of the two letters. In the Congruent condition, the action corresponds to the prompt (e.g., memoranda = AB, prompt refers to B, response = B); in the Incongruent condition, the action corresponds to the other item of the memoranda (e.g., memoranda = AB, prompt refers to B, response = A). After each trial, subjects inputted a rating regarding their urge to err on an eight-point scale, in which 1 signified “almost no urge” and 8 signified an “extremely strong urge.”

Subjects were instructed to respond to prompts as quickly and as accurately as possible. They pressed the semi-colon key (;) when responding to the letter “A” and the apostrophe key (’) when responding to the letter “B”. The mapping of keys to letters was fully counterbalanced across subjects. Each trial began with a ready prompt (?). Subjects, to indicate their readiness to commence the trial, pressed the space bar with their left hand. After a blank screen (300 ms) and a fixation cross (“+” for 500 ms), the memoranda were presented (1 s). Which memoranda were presented was randomly determined. This was followed by a delay period (4 s) that was followed by the prompt (3 s). In the Letter Prompt condition, a letter prompt appeared in the center of the screen. In the Dot Prompt condition, a filled circle (the prompt) appeared either above or below a horizontal line presented in the center of the screen (**Figure 1**). Subjects were instructed to respond to the prompt as quickly and as accurately as possible. In the Congruent condition, subjects were instructed to press the button which corresponded to the prompt. In the Incongruent condition, subjects were instructed to press the button which corresponded to the ‘other letter’ in the pair composing the memoranda.

After this response, subjects were asked to rate how strong their urge was to make a mistake, on an eight-point scale, in which 1 signifies “almost no urge” and 8 signifies an “extremely strong urge” (based on Morsella et al., 2009a,b). During the data collection for this pilot study, we took the opportunity to collect electroencephalographic data from the subjects. These pilot, neural data will not be discussed further. No such recordings occurred for the experiment (presented below) that was based on this pilot study.

Stimuli were presented in random order and care was taken to ensure that subjects responded an equal number of times to letters in either the top or bottom position. The experimental session consisted of 384 trials. These trials were divided into two large blocks (Dot Prompt versus Letter Prompt), each having 192 trials. Each of the two blocks contained miniblocks of Congruent and Incongruent trials (96 trials per miniblock). Because of data removal (discussed below) and the nature of the pseudo-randomization that was employed, the presentation order of the four kinds of blocks was not perfectly counter-balanced across subjects. This shortcoming is not featured in our experiment (presented below).

Once subjects completed the experiment, they completed a demographic form and responded to a series of funneled debriefing questions (following the procedures of Bargh and Chartrand, 2000), which included general questions to assess whether subjects (*a*) were aware of the purpose of the study, (*b*) had any strategies for completing the task, (*c*) had anything interfere with their performance on the task, and (*d*) tried their best to remember the letter pair that was presented at the beginning of the trial.

The data from four subjects were excluded from analysis, for the following reasons. For three of the subjects, the experimental software ceased to function, causing the experimental session to end prematurely. One of the subjects did not follow the instructions and pressed an incorrect button on all but one of the trials of the Dot Prompt condition. For the data from the remaining 29 subjects, based on the procedures of previous studies (Woodworth and Schlosberg, 1954; van Veen et al., 2001; Morsella et al., 2009a,b), we excluded from our analysis RTs less than or equal to 200 ms or greater than 2000 ms. For the RT analysis, we removed data from trials in which responses were inaccurate. This trimming method resulted in the loss of 502 (4.5%) out of 11,136 trials. No urge to err data were missing.

### Results

#### Urges to Err

In a fully within-subjects ANOVA with Congruence (Congruent versus Incongruent) as one factor and Prompt (dot versus letter) as the other factor, there was a main effect of Congruence, *F*(1,28) = 20.73, *p* < 0.0001 ($\eta_{p}^{2}$ = 0.43), in which urges were stronger for the Incongruent than the Congruent conditions, and a main effect of Prompt, *F*(1,28) = 15.57, *p* < 0.001 ($\eta_{p}^{2}$ = 0.36), in which urges were stronger for the dot prompt than the letter prompt (see descriptive statistic for all conditions in **Table 1**). There was no interaction between the two factors, *F*(1,28) = 0.50, *p* = 0.487. Planned comparisons revealed that all contrasts between the conditions were significant (*t*s > 2.9, *p*s < 0.05) except for that between Dot-Congruent and Letter-Incongruent, *p* = 0.63.

**Table 1:** Descriptive statistics for conditions of the Pilot Study and Experiment: means per condition with *SE* presented in parentheses.

	Urges to err	Error rates	Response times
**Pilot Study**			
Dot Congruent	1.67 (0.11)	0.02 (0.005)	569.34 (24.71)
Dot Incongruent	1.99 (0.14)	0.04 (0.01)	655.55 (28.68)
Letter Congruent	1.45 (0.13)	0.02 (0.01)	520.94 (19.98)
Letter Incongruent	1.71 (0.14)	0.04 (0.01)	603.54 (23.81)
**Experiment**			
Dot Congruent	1.66 (0.09)	0.04 (0.01)	679.03 (22.81)
Dot Incongruent	1.88 (0.10)	0.05 (0.01)	761.58 (23.14)
Letter Congruent	1.47 (0.08)	0.02 (0.003)	657.22 (24.46)
Letter Incongruent	1.67 (0.09)	0.04 (0.01)	666.12 (19.89)

#### Error Rates

Importantly, subjects were capable of performing this task: the mean proportion of errors across the 384 trials was 0.03 (*SD* = 0.029). Error rates were comparable across the four conditions (**Table 1**): in a fully within-subjects ANOVA with Congruence (Congruent versus Incongruent) as one factor and with Prompt (dot versus letter) as the other factor, there was no main effect of Congruence, *F*(1,28) = 2.36, *p* = 0.14, and no main effect of Prompt, *F*(1,28) = 0.002, *p* = 0.97. There was no interaction between the two factors, *F*(1,28) = 0.006, *p* = 0.94.

#### Response Times

In a fully within-subjects ANOVA with Congruence (Congruent versus Incongruent) as one factor and Prompt (dot versus letter) as the other factor, there was a main effect of Congruence, *F*(1,28) = 69.74, *p* < 0.0001 ($\eta_{p}^{2}$ = 0.71), in which RTs were longer for the Incongruent than the Congruent conditions, and a main effect of Prompt, *F*(1,28) = 7.93, *p* = 0.009 ($\eta_{p}^{2}$ = 0.22), in which RTs were longer for the dot prompt than the letter prompt (see descriptive statistics for all conditions in **Table 1**). There was no interaction between the two factors, *F*(1,28) = 0.019, *p* = 0.891. Planned comparisons revealed that all contrasts between the conditions were significant (*t*s > 2.2, *p*s < 0.05) except for that between Dot-Congruent and Letter-Incongruent, *p* = 0.13. The mean correlation between a subject’s RTs and urges, stemming from the 384 trials, was 0.51 (Fisher’s *r* to *z*, *p* < 0.001), suggesting that subjects may have based their judgments on observing their response times (see General Discussion).

### Discussion

The findings from our pilot study revealed that subjects are capable of performing this task, even though the task is more complicated than that of previous studies (e.g., Hubbard et al., 2013). On average, accuracy rates were above 90%. However, our pilot study suffered from two limitations. First, the letter prompt, unlike the dot prompt in the Dot condition, appeared always in the center of the screen. This difference in the nature of spatial location of the prompts in the Dot and Letter conditions renders it difficult to compare the effects of the two conditions. Second, the order of presentation of the blocks of trials was not counterbalanced fully across subjects. This was due in part to the fact that the data from some subjects were excluded from analysis.

## Experiment

Our experiment was based on previous research (e.g., Hubbard et al., 2013) and on our pilot study. Unlike in our pilot study, in the Letter Prompt condition, the letter prompt did not appear in the center of screen but in the location where, in the Dot condition, the dot would appear (i.e., above or below the horizontal line; **Figure 1**). In the Congruent Condition, the letter prompt was presented always in the location that corresponded to the location of the letter held in memory. Catch trials, in which the memoranda consisted of two identical letters, composed half of the 384 trials. The sample size for our experiment was more than double that of our pilot study.

### Subjects

San Francisco State University students (*n* = 64; females = 48; *M*_Age_ = 22.12, *SD* = 5.44) participated for course credit. The involvement of human subjects in our project was approved by the Institutional Review Board at San Francisco State University.

### Procedures

**FIGURE 2:**
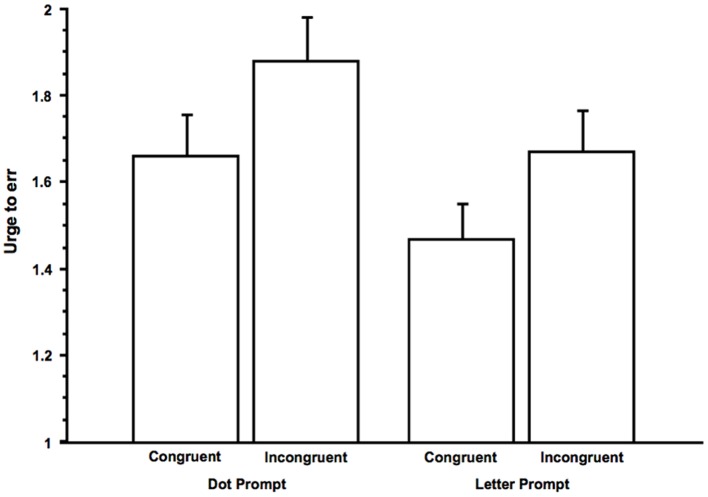
**Mean urges to err as a function of prompt and condition (congruent versus incongruent).** Error bars indicate *SEs*.

The procedures were identical to those of our pilot study except that (1) the letter prompt now appeared in the location where, in the Dot condition, the dot would appear (**Figure 1**); (2) the background of the screen was white; (3) all stimuli were presented in black font on an Apple iMac computer monitor (50.8 cm); stimulus presentation was controlled by PsyScope software (Cohen et al., 1993); (4) for the 384 trials, the order of presentation of the two large blocks (Dot prompt [192 trials] versus Letter prompt [192 trials]) and of the miniblocks (Congruent [96 trials] and Incongruent [96 trials]) were fully counterbalanced across subjects.

The data from eight subjects were excluded from analysis, for the following reasons. One of the subjects received a telephone call and, upon receiving it, terminated the experimental session. For two of the subjects, there was a malfunction regarding the experimental software, causing the experiment to end abruptly and prematurely. For one of the subjects, there was a programming error in the software script. Three of the subjects did not follow instructions and did not seem to understand what was asked of them. (The first of these participants, instead of performing the task, played with the chair; the second performed the Incongruent condition as if it were the Congruent condition; and the third was not attending to the computer screen.) Last, one of the subjects fell asleep during the experimental session. For the data from the remaining 64 subjects, the trimming method from our pilot study resulted in the loss of 1,553 (6.3%) out of 24,576 trials. Omitted responses and typing errors resulted in the loss of 338 (1.4%) out of 24,576 urge to err ratings.

#### Urges to Err

As illustrated in **Figure 2**, in a fully within-subjects ANOVA with Congruence (Congruent versus Incongruent) as one factor and Prompt (dot versus letter) as the other factor, there was a main effect of Congruence, *F*(1,63) = 25.99, *p* < 0.0001 ($\eta_{p}^{2}$ = 0.29), in which urges were stronger for the Incongruent than the Congruent conditions, and a main effect of Prompt, *F*(1,63) = 13.93, *p* < 0.001 ($\eta_{p}^{2}$ = 0.18), in which urges were stronger for the dot prompt than the letter prompt (see descriptive statistics for all conditions in **Table 1**). (A more conservative, Friedman test for the four kinds of conditions experienced by subjects was significant, *p* < 0.0001.) In the ANOVA, there was no interaction between the two factors, *F*(1,63) = 0.05, *p* = 0.82. The main effects of Congruence and Prompt were found also following Bonferroni correction, *p*s < 0.05. Planned comparisons revealed contrasts between the Dot-Incongruent condition and the Dot-Congruent condition, *t*(63) = 4.33, *p* < 0.0001, and between the Letter-Incongruent condition and Letter-Congruent condition, *t*(63) = 3.24, *p* < 0.01. These same contrasts between conditions was found in our pilot study and with the non-parametric, Wilcoxon signed-ranked test, *p*s < 0.0001.

In our experiment, there were three dependent measures: urges to err, accuracy, and RTs. Tests of normality (the Kolmogorov–Smirnov test) revealed that, for each of the four conditions (i.e., Dot-Congruent, Dot-Incongruent, Letter-Congruent, and Letter-Incongruent), the distribution of by-subject means did not violate the assumption of normality (Kolmogorov–Smirnov *p*s > 0.05). In addition, for each of these three dependent measures, the differences between the means of the Congruent and Incongruent conditions for the dot and letter prompts, too, did not violate the assumption of normality (Kolmogorov–Smirnov *p*s > 0.20). Nevertheless, for the sake of thoroughness and because some distributions of values appeared leptokurtic and highly skewed, and because, for three particular conditions (i.e., urges to err for the Letter-Congruent, and error rates for the Dot-Congruent condition and Letter-Congruent condition), the Kolmogorov–Smirnov *p*s did approach significance (*p*s = 0.06), we provide in this section, where appropriate and in addition to the results from our parametric tests, the results from comparable non-parametric tests, which are more conservative. We also provide information about the kurtosis and skewness of each distribution. For urges to err, the skewness of each distribution was: Dot-Congruent = 1.66, Dot-Incongruent = 1.00, Letter-Congruent = 2.15, and Letter-Incongruent = 1.91. For this same measure, the kurtosis for each distribution was: Dot-Congruent = 2.25, Dot-Incongruent = 0.22, Letter-Congruent = 4.38, and Letter-Incongruent = 3.54.

#### Error Rates

Again, as in the case of our pilot study, subjects were capable of performing the task: the mean proportion of errors across the 384 trials was 0.04 (*SD* = 0.028). See error rates for all conditions in **Table 1**. Unlike in our pilot study, error rates varied by condition: in a fully within-subjects ANOVA with Congruence (Congruent versus Incongruent) as one factor and Prompt (dot versus letter) as the other factor, there was a main effect of Congruence, *F*(1,63) = 22.88, *p* < 0.0001 ($\eta_{p}^{2}$ = 0.27), and a main effect of Prompt, *F*(1,63) = 11.76, *p* = 0.001 ($\eta_{p}^{2}$ = 0.16). (A Friedman test for the four kinds of conditions experienced by subjects was significant, *p* < 0.0001.) In the ANOVA, there was no interaction between the two factors, *F*(1,63) = 2.03, *p* = 0.16. The main effects of Congruence and Prompt were found also following Bonferroni correction, *p*s < 0.05. Planned comparisons revealed contrasts between the Dot-Incongruent condition and the Dot-Congruent condition, *t*(63) = 2.48, *p* < 0.05) and between the Letter-Incongruent condition and Letter-Congruent condition, *t*(63) = 3.99, *p* < 0.001. These same contrasts between conditions were obtained with the non-parametric, Wilcoxon signed-ranked test, *p*s < 0.01. For error rates, the skewness of each distribution was: Dot-Congruent = 2.21, Dot-Incongruent = 1.29, Letter-Congruent = 1.83, and Letter-Incongruent = 2.51. For this same measure, the kurtosis for each distribution was: Dot-Congruent = 5.23, Dot-Incongruent = 1.24, Letter-Congruent = 3.43, and Letter-Incongruent = 7.57.

#### Response Times

In a fully within-subjects ANOVA with Congruence (Congruent versus Incongruent) as one factor and Prompt (dot versus letter) as the other factor, there was a main effect of Congruence, *F*(1,63) = 30.80, *p* < 0.0001 ($\eta_{p}^{2}$ = 0.33), in which RTs were longer for the Incongruent than the Congruent conditions, and a main effect of Prompt, *F*(1,63) = 32.61, *p* < 0.0001 ($\eta_{p}^{2}$ = 0.34), in which RTs were longer for the dot prompt than the letter prompt (see descriptive statistic for all conditions in **Table 1**). (A Friedman test for the four kinds of conditions experienced by subjects was significant, *p* < 0.0001.) There was an interaction between the two factors, *F*(1,63) = 20.07, *p* < 0.001 ($\eta_{p}^{2}$ = 0.24). The main effects of Congruence and Prompt were found also following Bonferroni correction, *p*s < 0.05. Planned comparisons revealed contrasts between the Dot-Incongruent condition and the Dot-Congruent condition, *t*(63) = 7.16, *p* < 0.0001 (Wilcoxon signed-ranked test, *p* < 0.001), but no difference between Letter-Congruent and Letter-Incongruent, *t*(63) = -0.76, *p* = 0.45. Although the contrast between Letter-Congruent and Letter-Incongruent was non-significant in this experiment, it was a significant effect in our pilot study. For RTs, the skewness of each distribution was: Dot-Congruent = 0.31, Dot-Incongruent = 0.03, Letter-Congruent = 0.60, and Letter-Incongruent = 0.28. For this same measure, the kurtosis for each distribution was: Dot-Congruent = -0.54, Dot-Incongruent = -0.55, Letter-Congruent = -0.47, and Letter-Incongruent = -0.63.

#### Correlational Analyses

The mean correlation between a subject’s RTs and urges, stemming from the 384 trials, was 0.37 (Fisher’s *r* to *z*, *p* < 0.01), suggesting that subjects may have based their judgments on the observations of their response times (see General Discussion). Having a sample size much larger than that of our pilot study allowed us to examine with confidence the correlation coefficients, between RT and urges to err, per condition: Dot-Congruent (*r* = 0.38), Dot-Incongruent (*r* = 0.45), Letter-Congruent (*r* = 0.35), Letter-Incongruent (*r* = 0.40). Fisher’s *r* to *z* revealed that, given the number (*n* = 96) of observations per condition, each of these coefficients, which resembled that of the coefficient found when we collapsed across conditions, is significant, *p*s < 0.05. Regarding the relationship between accuracy and urges to err, it was not the case that, if a subjects’ error rate was high, his or her mean urge ratings would vary proportionally, *r* = 0.18, *p* = 0.16. This lack of a correlation between mean accuracy and mean error rate must be interpreted only cautiously, as error rates were very low, which, for a correlational analysis, could introduce a restriction of the range.

## General Discussion

Our primary aim was to investigate the under-explored subjective (and metacognitive) aspects of working memory performance. With this aim, we built on investigations that have begun to examine the nature of the subjective, metacognitive states associated with different kinds of response interference (Mayr et al., 2003; Mayr, 2004; Morsella, 2005; Mulert et al., 2005; Rosen et al., 2007; Corallo et al., 2008; Morsella et al., 2009b) and developed a new experimental paradigm that involves working memory and subjective data (urges to err) on a trial-by-trial basis. Our primary goal was to develop a paradigm that subjects are capable of performing and in which (*a*) subjects do not know which response to execute before being presented with a go cue, (*b*) subjects do not know which information from the memoranda will be action-relevant, and (*c*) the association between the retrieval cue and the part of the memoranda to be acted upon is not straightforward and requires the application of a complex rule held in prospective memory. Developing such a new paradigm, independent of the potential findings it could be used to obtain, is an important contribution in its own right.

As an initial foray involving a new paradigm and an uncharted area of research, our project demanded conservative predictions and humble conclusions. First, and of most importance, the error rate data revealed that subjects are capable of performing this task, even though this task is more complex and difficult than previous tasks (e.g., Hubbard et al., 2013): accuracy rates in our paradigm were greater than 90%. Because of the catch trials, subjects could not simply apply the strategy of, in the Incongruent condition, making a response opposite of that indicated by the prompt. To obtain high accuracy rates in this paradigm, it is mandatory for the entire memoranda to be retrieved from memory.

As predicted, the subjective data revealed an effect of the factor Congruence, in which urges to err were strongest for incongruent trials. In our experiment, Congruence produced the predicted effect both when the prompt was a letter and when the prompt was not a letter (the Dot condition), suggesting that the effect of Congruence in the Letter Prompt Condition did not reflect only effects of (*a*), in the Incongruent condition, the letter prompt priming a motor response that happened to be incompatible with that of the intended, memory-based response, or (*b*), in the Congruent condition, the letter prompt incidentally priming the response associated with it. In addition, finding an effect from Congruence in both the Dot and Letter conditions suggests that the effect was not simply an artifact of subjects applying the strategy of, in the Congruent condition, completely disregarding the memoranda and attending only to the prompts. The factor Prompt (Letter versus Dot) led to subjective effects in which urges to err tended to be stronger for the Dot condition than the Letter condition. Understanding the nature of this effect will require further investigation.

Although urges to err correlated with RTs, the patterns of results found with the two kinds of data did not always mirror each other. In prior research, it has been found that, at times, trial-by-trial urges to err are more sensitive to experimental manipulations than are RTs (Morsella et al., 2011), just as RTs are often more sensitive to experimental manipulations than are error rates (e.g., in the Stroop task). In the present experiment, for the Letter condition of our experiment, there was no effect of Congruence on RTs, but subjective effects (e.g., urges to err) did arise systematically from this experimental manipulation. (A congruence effect on RTs was found, however, in the pilot study.) Such interesting dissociations between subjective and behavioral data have been found in previous studies involving trial-by-trial subjective measures (Morsella et al., 2009b). The dissociations discovered in our experiment require replication and further investigation.

In light of the present findings, it is important to appreciate that there is an important difference between trial-by-trial questions regarding task difficulty and questions about the kinds of fleeting urges that our subjects experienced. We have learned from previous endeavors that some subjects construe these two questions as pertaining to separate and distinct phenomena. For example, questions about task difficulty are about *the task* and not necessarily about the subjects’ just-experienced subjective state (i.e., the urge). In the present project, we queried about trial-by-trial urges because doing so replicates trial-by-trial measures used in previous studies involving response interference (e.g., Morsella et al., 2009b), and because, if the question were instead about task difficulty, some subjects might perceive the question as concerning, not his or her just-experienced subjective state, but rather about (a) how most people would perceive the difficulty of the task or (b) the difficulty of the task in terms of the nature of, not one’s actual experience, but properties of the task (e.g., the number of mental operations that the task demands).

As with all forms of self-report data, it is challenging to verify what subjects are introspecting at the moment when they are making their judgments. Self-report judgments are subject to memory distortions, even when they are made moments after the critical event (Block, 2007). One could question whether subjects in our pilot study and experiment were actually experiencing conscious urges. However, given the systematic changes in urges to err as a result of condition, it seems unlikely that subjects did not experience urges, did not follow instructions, and provided ratings only because of, say, experimental demand. This conclusion could be further corroborated by coupling our new paradigm with neuroimaging technologies. Such technologies would allow one to detect neural markers of conscious urges (see relevant evidence in Gray et al., 2013). It is important to note that neuroimaging evidence corroborates that, in other paradigms, subjects are in fact accurate about reporting the incidence of conscious mental contents (cf., Logothetis and Schall, 1989; Wyland et al., 2003; Mason et al., 2007; Mitchell et al., 2007; McVay and Kane, 2010).

Subjects’ judgments may be influenced by an overall sense of difficulty, which may be introspected directly or indirectly from inferences based on, for example, RT performance. In short, it is difficult to rule out an influence on judgments of processing speed or a general sense of effort (or any combination thereof). Nevertheless, as mentioned above, trial-by-trial subjective effects are unlikely to reflect solely an artifact of subjects observing their own RTs, for the effects arise even when the influence of RTs is statistically accounted for, and also when RT effects are eradicated (e.g., by having subjects delay their response; Morsella et al., 2009b). In addition, these theoretically predicted subjective effects are present when subjects merely sustain incompatible intentions (e.g., to point left and right) in a motionless state in which no response is emitted (Morsella et al., 2009a). Last, it is worth reiterating that, though subjects’ post-error corrections in interference paradigms lead, on a subsequent trial, to improved performance (e.g., faster RTs), reported urges to err actually increase in such trials (Etkin et al., 2010; Gyurak et al., 2011)—a contrast that has been explained as a dissociation between implicit measures of performance (e.g., RT) and explicit measures (e.g., self-reports about task difficulty; Etkin et al., 2010; Gyurak et al., 2011).

Nevertheless, because of the limitations inherent in introspection paradigms, we cannot rule out conclusively that ratings were based on self-observations involving RT performance or on strategies. Regarding the latter, subjects may have based their judgments on folk beliefs regarding how one should comport oneself in an experiment involving congruent and incongruent conditions. For example, perhaps subjects based their judgments on heuristics such as, “If I am in the Incongruent condition, then I will always report 6 as the rating; if I am in the Congruent condition, then I will always report 1 as the rating.” Although, this cannot be fully ruled out by the present data, this alternative seems unlikely given that subjects’ ratings tended to vary across trials within each condition. For instance, for the Congruent Dot condition, the first seven ratings from a subject selected at random were *1*, *1*, *1*, *7, 1, 1*, and *8*. Another subject yielded the following sequence of ratings for a sequence of six trials in the Letter Incongruent condition: *1*, *1*, *2*, *1*, *4*, and *3.* Of course, it may well be that subjects were using a more sophisticated heuristic when engendering these current data (Morsella et al., 2009b).

Future investigations on the subjective aspects of working memory performance are necessary to qualify the kinds of conclusions that can be drawn from these initial introspection-based data. Future research could also focus on the nature of the conscious imagery, about the memoranda, during the delay period. Such experiments might benefit from having a delay period that is longer and from having manipulations in which the amount of information held in the memoranda is varied experimentally, as in Jantz et al. (2014). Regarding the former, in our experiment, the delay period was too short (4 s) for subjects to be able to indicate conscious imagery by button press. Regarding the latter, with the current version of our new task, it is unlikely that a memory load would arise from a memoranda consisting of only two items.

## Conclusion

The findings from our experiment reveal that (*a*) subjects can successfully perform this task, (*b*) reliable subjective and behavioral data can be obtained on a trial-by-trial basis, and (*c*) the subjective effects associated with this working memory task are systematic, measurable, and arise from processing in a principled fashion. We hope that our novel paradigm will serve as a foundation for future experimental projects on the relationship between working memory performance and consciousness—an under-explored nexus whose investigation is likely to reveal many insights about working memory, consciousness, and metacognition.

## Author Contributions

SB: conducted experiment 2; AG: conducted the pilot study; EM: idea for the paradigm; MG and AV: helped with the design and data collection; all authors contributed to the manuscript.

## Conflict of Interest Statement

The authors declare that the research was conducted in the absence of any commercial or financial relationships that could be construed as a potential conflict of interest.

## Footnotes

1 The terms *working memory* and *response interference* are defined below.

2 It is worth mentioning here that subjects can perform successfully a variant of the classic flanker task in which, on some trials, subjects must perform an action that is the opposite of that indicated by the target (Velasquez and Morsella, 2016). In Velasquez and Morsella (2016), this action is prompted by a cue (e.g., a dot presented beneath the target) that is presented in addition to the flankers.
